# Estimation of River Pollution Index in a Tidal Stream Using Kriging Analysis

**DOI:** 10.3390/ijerph9093085

**Published:** 2012-08-29

**Authors:** Yen-Chang Chen, Hui-Chung Yeh, Chiang Wei

**Affiliations:** 1 Department of Civil Engineering, National Taipei University of Technology, Taipei 10608, Taiwan; Email: yenchen@ntut.edu.tw; 2 Department of Natural Resources, Chinese Culture University, Taipei 11114, Taiwan; Email: hcyeh@faculty.pccu.edu.tw; 3 Experimental Forest, National Taiwan University, Jhu-Shan, Nantou 55750, Taiwan

**Keywords:** geostatistics, kriging, river pollution index, tidal stream, water quality

## Abstract

Tidal streams are complex watercourses that represent a transitional zone between riverine and marine systems; they occur where fresh and marine waters converge. Because tidal circulation processes cause substantial turbulence in these highly dynamic zones, tidal streams are the most productive of water bodies. Their rich biological diversity, combined with the convenience of land and water transports, provide sites for concentrated populations that evolve into large cities. Domestic wastewater is generally discharged directly into tidal streams in Taiwan, necessitating regular evaluation of the water quality of these streams. Given the complex flow dynamics of tidal streams, only a few models can effectively evaluate and identify pollution levels. This study evaluates the river pollution index (RPI) in tidal streams by using kriging analysis. This is a geostatistical method for interpolating random spatial variation to estimate linear grid points in two or three dimensions. A kriging-based method is developed to evaluate RPI in tidal streams, which is typically considered as 1D in hydraulic engineering. The proposed method efficiently evaluates RPI in tidal streams with the minimum amount of water quality data. Data of the Tanshui River downstream reach available from an estuarine area validate the accuracy and reliability of the proposed method. Results of this study demonstrate that this simple yet reliable method can effectively estimate RPI in tidal streams.

## 1. Introduction

The complex flow of tidal streams is mainly influenced by interactions between river water and seawater. Thus, tidal streams are in constant flux as they adapt to river and climate conditions. The half-day tidal variation of the sea is the main driver of cyclic fluctuation in tidal streams [1]. Seasonal changes in flow conditions from estuaries determine water salinity. Upstream flooding is a factor in the changes in the vertical texture of water bodies and is attributed to flow irregularities. Among climatic factors, wind conditions significantly affect tidal streams. Waves caused by wind shear alter the circulation patterns and mixing process of river water and seawater. Within this process, only a 2% difference in density between river water and sea water results in a horizontal pressure gradient, which affects water flow. This difference is mainly attributed fluctuations in temperature and water salinity; the role of the latter is significantly stronger [2]. The physical process of the flow mechanism appears to be complex and is rather difficult to explain. Moreover, this phenomenon drives additional processes in the water body such as sedimentology, biology, and chemistry [3]. Thus, traditional models fail to accurately estimate the flow and water quality of tidal streams. The flow field must be estimated through a hydraulic model before determining water quality. Therefore, a considerable amount of river data including sectional features, flow, level, and quality is necessary to calibrate the numerous model parameters and requires substantial time, labor, and capital [4,5,6].

The application of geostatistical methods or those combined with other models to water quality monitoring and estimation has been discussed extensively during the past two decades. Lo *et al.* (1999) [7] applied a steady-state quality model to simulate the biochemical oxygen demand (BOD) and kriging theory for selecting optimal sampling locations and frequency. Their results indicated a total number of monitoring stations in the Keelung River of 21 and a sampling frequency of approximately 2–3 times per month. Mohammad *et al.* [8] also applied genetic algorithm-, kriging- and analytical hierarchy process-based methods to evaluate suitable sampling locations and frequency in Iran. Yang *et al.* [9] proposed a spatial regression method in conjunction with the kriging approach to estimate the nitrogen concentrations of nonpoint source pollution (NOP) in some Iowa (USA) streams. Polus *et al.* [10] demonstrated that geostatistical methods reduce uncertainties in a physically based models (DPBMs) distributed along the Seine River in France. Liu *et al.* [11] used hierarchical clustering analysis, principal component, and factor analysis with geostatistics to assess the water quality of an alpine lake in Taiwan. Moreover, other researches focused on spatial modeling of the scaling issue. Militino *et al.* [12] proposed a linear mixed incorporating both spatial as well as longitudinal information for detecting excessive nitrate. Garreta *et al.* [13] proposed various relevant methods based on geostatistics for application to prediction and error maps of Meuse and Moselle basins in France. 

Tidal stream water quality is difficult to simulate with the water quality model because of the many effects of run-off convergence from rainfall upstream and tide recession downstream; thus, a geostatistical method was used in this study. Four variables of water quality were obtained simultaneously from sampling stations along the tidal streams. The water quality for each station was then estimated by kriging analysis to evaluate the pollution of tidal streams. In particular, our proposed algorithm is based on 1D kriging and provides a simple and efficient solution for the complexities of boundary conditions encountered in traditional 2D hydrological models.

## 2. Theory and Methods

The geostatistical method adopted in this study for estimating RPI is based on the sampling data obtained from the Tanshui River. The use of an RPI is characterized by the fact that, although the spatial distribution of drainage area is 2D, pollution in the mainstream generally remains in a 1D variable flow transmitted from upstream to downstream. Thus, spatial estimation based on a 2D random variable domain is impossible. In this study, RPI calculations were performed at various separate sampling points in the mainstream of the Tahan River upstream from the converging point between the Hsintien and Keelung rivers to establish the testing semivariogram by geostatistics on an hourly basis. On the basis of the optimal theoretical semivariogram model, the RPI values were then estimated hourly for other areas without measuring points along the three rivers. Since the Tanshui River is a tidal stream, pollutants are likely transmitted back upstream at high tide. The overlapping estimates of RPI per river kilometer among the three rivers were averaged; data from the separate stations were estimated directly without overlapping. 

### 2.1. Kriging Analysis

Geostatistics, a scientific method that analyzes spatial structures, is based on parameters of natural phenomena in the structural characteristics of spatial distribution. In this method, regionalized variables are established in various locations, the estimation of which is based on variograms. Various research fields that apply the theory of regionalized variables include meteorology, soil physics, groundwater, mining and metallurgy, environmental monitoring, and hydrology [14,15,16,17,18].

Matheron [19,20] pioneered kriging analysis, referring to the function as Krige. The spatial variation of rainfall was interpolated by using an ordinary kriging method. While not designed to optimize the appearances of interpolation, kriging is characterized by its statistical capability to increase estimation accuracy at grid points. Kriging is the decomposition of the variable Z(x) into the sum:


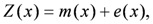
(1)


where *m*(*x*) represents the mean and *e*(*x*) represents the zero-mean function specific for a given position x. Notably, mean *m*(*x*) is an unknown constant that leads to ordinary kriging, which follows the best linear unbiased estimator. The kriging estimator of derived as if all n observed data used is of the linear model as the form:


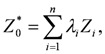
(2)


where *Z_i_* represents observed data and *λ_i_* represents a weight placed on *Z_i_*. Using the unbiased estimator,


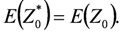
(3)

To satisfy the optimal condition, *λ_i_* is selected to minimize the error *Z*_0_ – *Z*_0_^*^:


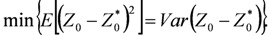
(4)

Equation (4) can be solved by the method of Lagrange multipliers, subsequently yielding:


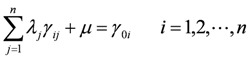
(5)



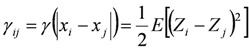
(6)



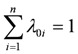
(7)


where *γ_ij_* represents the covariance of *i* and *j*; |*x_i_* − *x_j_*| represents the distance between *x_i_* and *x_j_*; *μ* is the mean value.

The kriging variance (*σ_ok_^2^*), which provides a measure of the error associated with the kriging estimator, is obtained by premultiplying the first *n* equation of (5) by *λ_i_*:


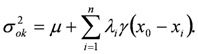
(8)


Based on the hypothesis of second-order stationarity, the development of kriging assumes that the mean and variogram are known. Therefore:


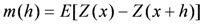
(9)



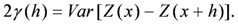
(10)


**Figure 1:**
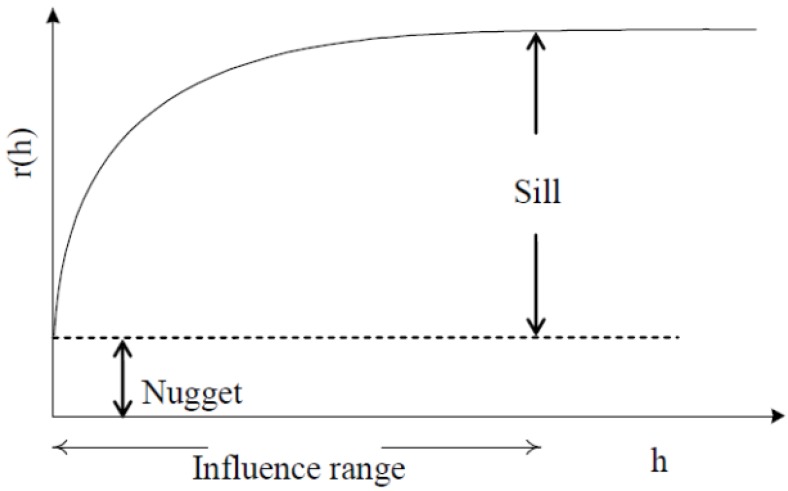
A diagram of the theoretical semivariogram.

**Figure 2:**
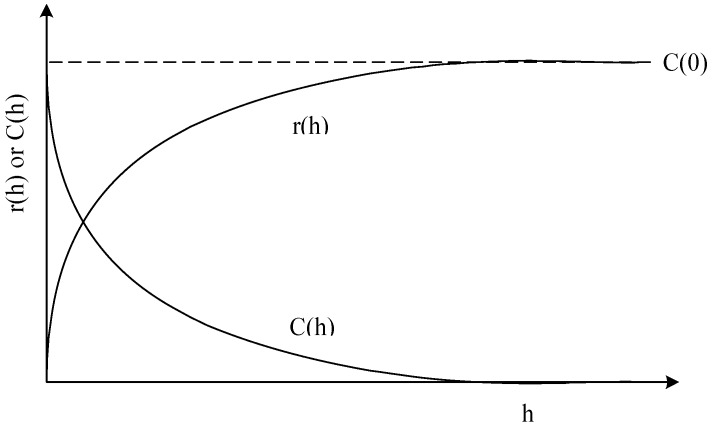
A diagram of the semivariogram r(h) and covariance function C(h).

**Figure 3:**
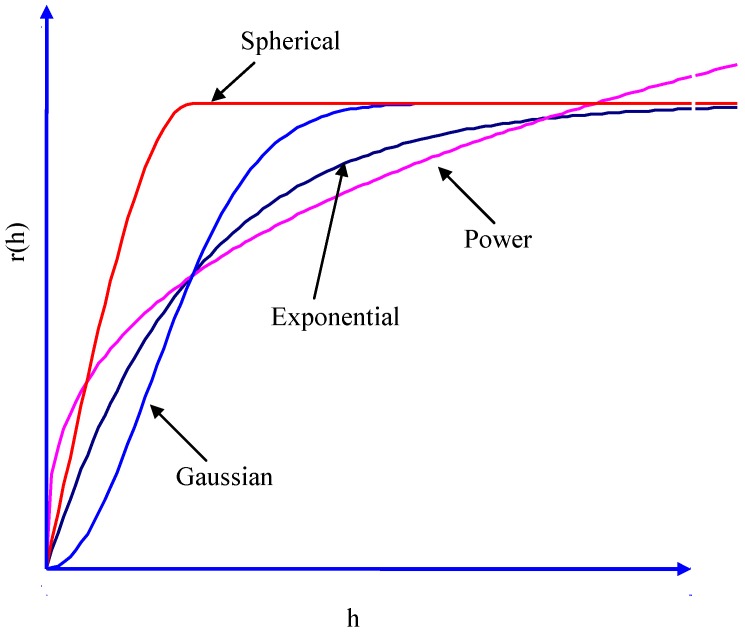
The theoretical semivariogram.

Variance of the increments has a finite value 2*γ*(*h*), depending on length *h* within the domain. The variogram indicates the extent of which the dissimilarity between *Z*(*x*) and *Z*(*x + h*) evolves with distance *h*. The graph of *γ*(*h*) against *h* reveals that the semivariogram increases with *h*, as shown in Figure 1. However, the semivariogram is bounded by a finite value known as sill. Notably, *Z*(*x*) and Z(*x *+ *h*) are uncorrelated with each other when *h* is larger than sill. A nugget effect may occur when significant variance occurs in a very short distance *h*. Additionally, the semivariogram and covariance function shown in Figure 2 are related. The value of *γ*(*h*) approaches *C*(0) when distance *h* increases to infinity. Several models, including spherical, exponential, Gaussian, and power-law models, are used to correlate with the relation of *γ*(*h*) and *h* to determine the sill and range (Figure 3). The power model is expressed as:


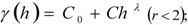
(11)


the spherical model:


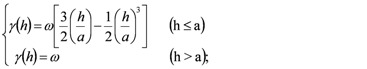
(12)


the exponential model:


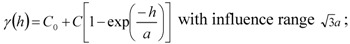
(13)


and the Gaussian model:


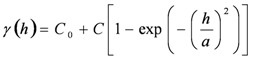
(14)


The exponential mode is a conventionally used covariance function for modeling discontinuity at the origin of the variogram. In addition to the four basic theoretical models described above, a nested structure consisting of these models can be used to correlate with the realistic variance of a random field. Additional details of the kriging theory were reported by Journel and Huijbregts [21].

### 2.2. River Pollution Index

The conventionally adopted classification system in Taiwan for monitoring water quality is an RPI [22] that includes four variables: dissolved oxygen (DO), biochemical oxygen demand (BOD_5_), suspended solids (SS), and ammonia nitrogen (NH_3_-N). DO is an important index for the quality of water bodies and includes dissolutions from the atmosphere, natural and artificial aeration, and photosynthesis from water plants. In water contaminated by organic matter, DO is consumed by aquatic microorganisms during decomposition; hypoxia occurs when DO in the water is diminished. BOD_5_ indicates the content of organic matter that can be decomposed by aquatic microorganisms, indirectly representing the degrees of contamination by organic matter in water bodies. Organic matter containing nitrogen is derived mainly from the decomposition of animal waste, animal corpses, and plant remains. During the decomposition process, amino acids are released first, followed by the sequential release of ammonia nitrogen, nitrite nitrogen, and nitrate nitrogen until stabilization. Therefore, the presence of ammonia indicates the short-term contamination of the water body. SS refer to organic or inorganic particles suspended in water by stirring or flowing, including colloids. SS impair light penetration in the water, and their effects on aquatic organisms are similar to those of turbidity. SS deposited on riverbanks block water flow, while solids deposited in reservoir areas diminish reservoir capacity. 

Each variable of water quality used to determine RPI is converted to one of four index scores (*S_i_* = 1, 3, 6, or 10). Notably, RPI refers to the arithmetic average of these index scores with respect to the water quality:


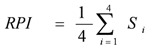
(15)


where *S_i_* represents the index scores based on Table 1 and the RPI value ranges from 1 to 10. According to the river pollution index listed in Table 1, the four classifications of pollution are unpolluted, negligibly polluted, moderately polluted, and severely polluted. 

**Table 1:** Definition of river pollution index (RPI).

Items	Ranks			
	Unpolluted	Negligibly polluted	Moderately polluted	Severely polluted
DO (mg/L)	Above 6.5	4.6–6.5	2.0–4.5	Under 2.0
BOD_5_ (mg/L)	Under 3.0	3.0–4.9	5.0–15	Above 15
SS (mg/L)	Under 20	20–49	50–100	Above 100
NH_3_-N (mg/L)	Under 0.5	0.5–0.99	1.0–3.0	Above 3.0
Index Scores (*S*_i_)	1	3	6	10
RPI	Under 2	2.0–3.0	3.1–6.0	Above 6.0

## 3. Study Site Descriptions

A length of 159 km and drainage area of 2,726 km^2^ makes the Tanshui River the third largest river in Taiwan. The Tahan, Hsintien, and Keelung rivers constitute the three main tributaries converging around Taipei from south to north. The Tahan River originates from Pintian Mountain at 3,529 m above sea level and flows through Hsinchu, Taoyuan and Taipei via the Shihmen Reservoir with a drainage area of 1,200 km^2^. The Tanshui River begins at Jiangzicui and converges with the Tahan and Hsintien rivers; its mainstream converges with the Keelung River at Guandu, flowing through Danshui into the Taiwan Strait. The drainage area of the Hsintien River is approximately 900 km^2^, and its upstream consists of the Beishih and Nanshih rivers. Two springs of the Beishih River originate near Kanchenkang at an elevation of approximately 620 m; the other begins near Ping Qi at an elevation of approximately 700 m. Two sources converge at the crossing and disembogue into Feitsui Reservoir. Nanshih River originates at the northern Chilan Mountain and flows northbound at an elevation of approximately 2,130 m.

The Beishih and Nanshih rivers converge near Hsintien, then discharge into the Tanshui River at Jiangzicui. The Keelung River originates at Jingtong Mountain with gorges above Badu and flows downward into a plain to converge with the Tanshui River at Guandu, which has a drainage area of 600 km^2^.

**Figure 4:**
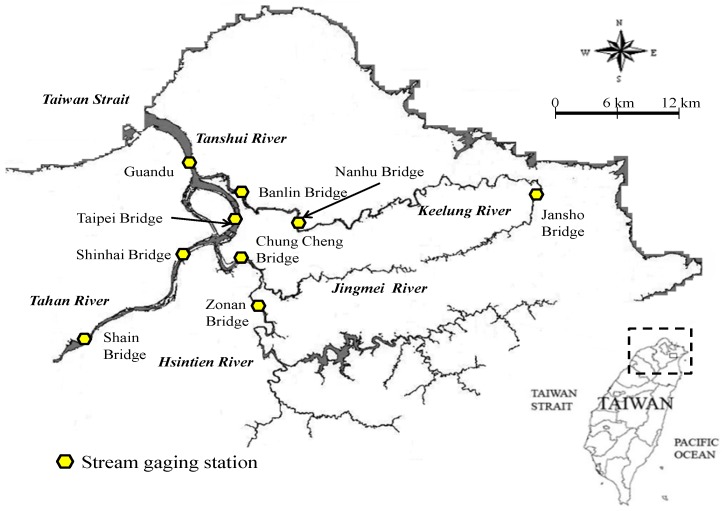
Map of study area.

This study analyzed water quality data from nine sampling stations along the drainage area of the Tanshui River at the Shain and Shinhai bridges along Tahan River; the Zonan and Chung Cheng bridges along the Hsintien River; the Jansho, Nanhu, and Banlin bridges along the Keelung River; and the Taipei and Guandu bridges along the Tanshui River (Figure 4). The data included DO, BOD_5_, NH_3_-N, and SS values of water quality obtained from the sampling stations during 13 h from 5 a.m. to 5 p.m. on 29 September 2010. Each data point was converted to the corresponding score index; indicator integral values were later calculated by consolidating the data of the four categories. The results represent the RPI for each sampling point.

## 4. Results and Discussion

### 4.1. One-Dimensional Design along the Tanshui River

**Figure 5:**
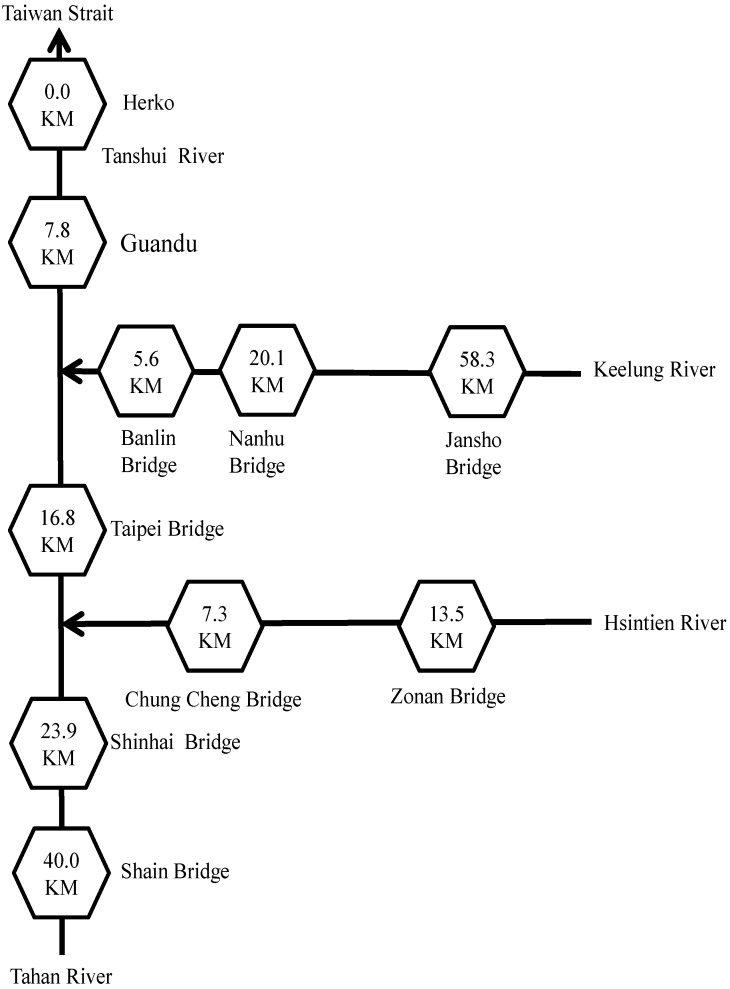
Distances in river kilometers of sampling stations in the catchment of the Tanshui River.

1D ordinary kriging analysis was performed along the river to estimate RPIs of the Tanshui River during a 13 h period. First, this study divided the drainage area of the Tanshui River into three sections. The first section included four sampling stations along the Keelung and Tanshui rivers: the Jansho, Nanhu, Banlin and Guandu bridges from upstream to downstream. The second section included four sampling stations along the Hsintien and Tanshui rivers: the Zonan, Chung Cheng, Taipei, and Guandu bridges from upstream to downstream. The third section included four sampling stations along the Tahan and Tanshui rivers: the Shain, Shinhai, Taipei, and Guandu bridges from upstream to downstream. The 1D distance between two sampling stations equaled the distance from an estuary along the river direction. Figure 5 describes the spatial relative positions and distance of the rivers in the Tanshui River drainage area. The results of the four water quality designations including DO, BOD_5_, SS, and NH_3_-N are listed in Table 2. 

### 4.2. Spatial Variability Analysis

The 1 D ordinary kriging analysis was performed along the river to estimate RPI. The hourly testing semivariogram for the three rivers in the Tanshui River drainage area were calculated, and the data of the testing semivariograms were mixed. The results obtained were then applied to the theoretical semivariogram models, including power, sphere, index, and Gaussian models. Finally, RPIs of the Tanshui River were estimated by using the theoretical semivariogram model. Individual semivariograms were established for three estuaries. 

**Table 2:** Results of water quality sampled from nine sites and their computed RPI values.

Water quality	DO (mg/L)			BOD_5_ (mg/L)			NH_3_-N (mg/L)			SS (mg/L)			RPI		
Station	mean ± std	Min.	Max.	mean ± std	Min.	Max.	mean ± std	Min.	Max.	mean ± std	Min.	Max.	mean ± std	Min.	Max.
Shain Bridge	4.45 ± 0.84	2.8	6.1	2.21 ± 0.33	1.7	2.6	0.02 ± 0.01	0.01	0.04	13.76 ± 3.91	10.1	23.1	1.98 ± 0.43	1.50	2.75
Shinhai Bridge	1.22 ± 0.77	0.1	2.8	8.45 ± 2.77	4.3	12.7	5.72 ± 0.94	4.37	7.40	32.08 ± 6.55	23.2	44.2	7.04 ± 0.54	5.50	7.25
Zonan Bridge	3.39 ± 0.43	2.7	4.0	1.89 ± 0.58	1.3	2.8	0.53 ± 0.37	0.13	1.25	17.39 ± 5.00	7.0	24.5	2.71 ± 0.42	2.25	3.50
Chung Cheng Bridge	4.07 ± 0.75	2.9	5.1	2.48 ± 0.61	1.5	3.7	1.58 ± 0.44	0.82	2.41	23.88 ± 10.32	13.9	54.0	3.67 ± 0.59	2.75	4.50
Jansho Bridge	3.99 ± 0.46	3.1	4.8	1.20 ± 0.15	1.0	1.4	0.01 ± 0.01	0.01	0.03	13.95 ± 9.48	3.4	28.9	2.38 ± 0.26	2.00	2.75
Nanhu Bridge	3.32 ± 0.57	2.4	4.2	3.11 ± 0.81	1.8	4.5	0.70 ± 0.22	0.37	0.96	38.94 ± 26.07	15.8	95.8	3.52 ± 0.81	2.25	4.50
Banlin Bridge	1.76 ± 0.22	1.4	2.1	2.98 ± 0.59	2.2	4.1	1.98 ± 0.14	1.64	2.16	13.45 ± 2.88	10.5	18.9	4.50 ± 0.46	3.50	5.00
Taipei Bridge	1.78 ± 0.38	1.4	2.6	2.98 ± 0.96	1.6	4.4	3.73 ± 0.71	2.55	5.33	30.51 ± 14.75	14.2	61.5	5.88 ± 1.12	3.50	7.25
Guandu Bridge	2.27 ± 0.34	1.5	2.7	1.96 ± 1.49	0.01	6.1	1.66 ± 0.44	0.53	2.21	20.81 ± 6.09	12.5	33.2	3.96 ± 0.67	2.75	5.25

**Figure 6:**
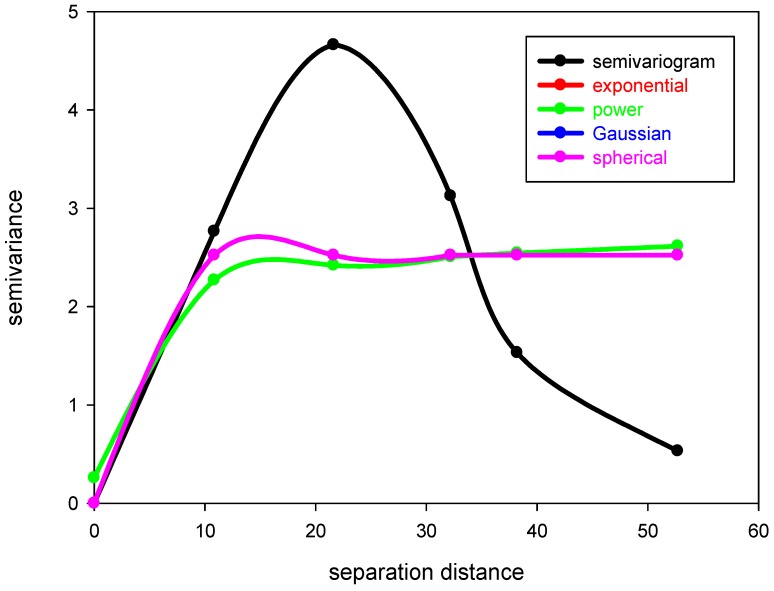
Fitted diagram of experimental and theoretical semivariograms of data obtained at 5 a.m. on 29 September 2010.

The RPI obtained at 5 a.m. on 29 September 2010, from the Tanshui River was chosen as the standard value. In this study, subsequent testing semivariograms were applied to the theoretical semivariogram models. Table 3 and Figure 6 summarize the results applied to the Tanshui River. These error sums of squares results are expressed as RSS; a smaller value implies a smaller error. This study demonstrated that the highest error sum of squares occurred in the power model, while the applied results from the other models were similar. Additionally, the correlation coefficient of the regression model is expressed as R^2^; a higher coefficient implies better applied results. According to Table 3, the coefficients of determination from the sphere, index, and Gaussian models were higher than those of the power model. An inflection phenomenon occurred in the Gaussian model at the short-distance area, while the sphere model was limited to certain distances. Therefore, in this study, the index model was selected to calculate the RPIs for those rivers. Table 4 summarizes the applied results of the index model for the 13 h of study along the Tanshui River.

**Table 3:** Parameters of the four fitted theoretical semivariograms.

Parameter	Power	Exponential	Gaussian	Spherical
*C*_0_	−0.005	−135.409	0.001	−0.006
*c*	2.318	136.996	2.312	2.320
*a*	0.417	0.002	1.000	0.480
Least Error Sum of Squares (RSS)	3.968	4.558	3.968	3.968
Coefficient of Determination (R^2^)	0.5289	0.4589	0.5289	0.5289

**Table 4:** Fitted parameters of exponential model.

Time 29 September 2010	*C*_0_	*C*	*a*	RSS	R^2^
5 a.m.	−0.005	2.528	0.420	9.949	0.3476
6 a.m.	−0.003	2.300	0.528	6.114	0.4181
7 a.m.	−0.006	2.773	0.424	16.512	0.2787
8 a.m.	−0.005	2.318	0.417	3.968	0.5289
9 a.m.	−0.007	3.549	0.437	11.234	0.4818
10 a.m.	−0.002	2.215	0.617	6.567	0.3829
11 a.m.	−0.004	3.355	0.631	18.835	0.3317
noon	−0.002	2.003	0.607	5.725	0.3678
1 p.m.	−0.015	3.201	5.662	6.497	0.5555
2 p.m.	−0.002	2.073	0.623	11.562	0.2174
3 p.m.	−0.002	1.906	0.692	4.571	0.3727
4 p.m.	−0.002	1.609	0.605	4.969	0.3020
5 p.m.	−0.002	2.221	0.609	9.146	0.3095

### 4.3. Estimation of RPI

This study used 1D ordinary kriging analysis to examine the RPI of the Tanshui River. During the study, four values of water quality obtained from the sampling stations were assigned to the corresponding points; the RPIs at various time intervals were calculated for each sampling station. Finally, 1D ordinary kriging analysis was performed again to estimate the RPIs along the Tanshui River. As shown in Figure 5, RPIs were individually estimated for three estuaries from Herko to upstream points. Hence, the computation for RPIs of the main estuary of the Tanshui River can be divided into three sections. The first section, from its origin to its convergence with the Hsintien River, included the same RPI as that estimated for the Tanshui River itself. In the second section, between the connections with the Hsintien and the Keelung rivers, the RPI was the average of that estimated for the Tanshui and Hsintien rivers. In the third section, between the convergence with the Keelung River and Herko, the RPI was equal to the average of all three RPI estimates. 

**Figure 7:**
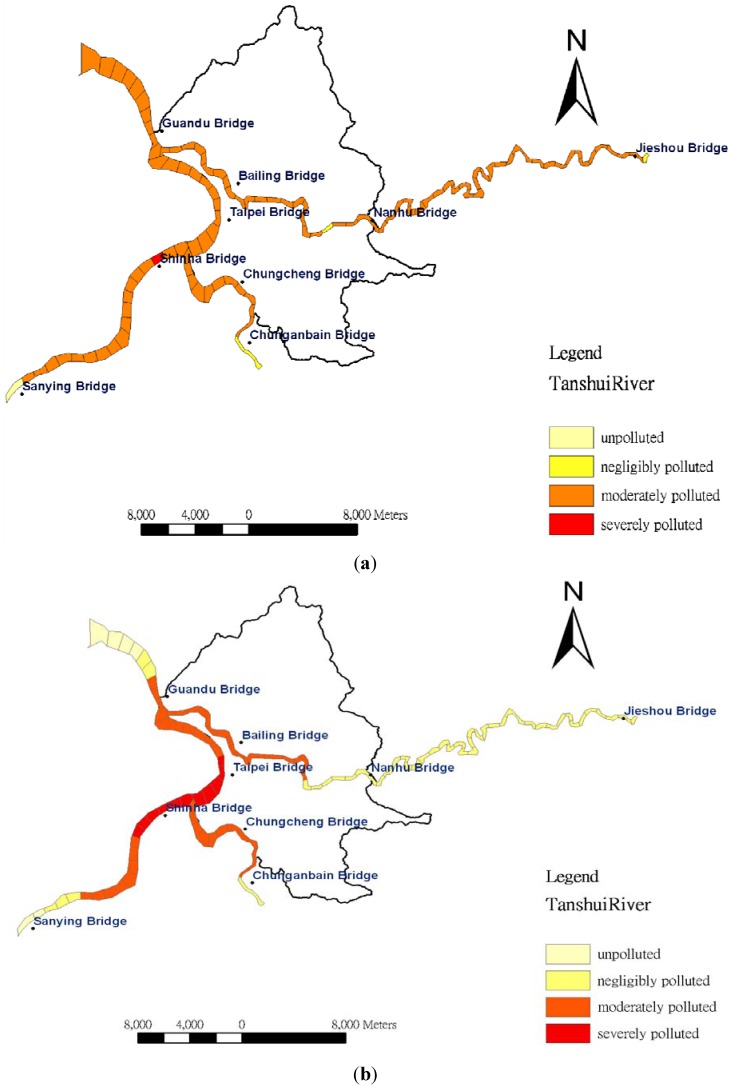
Spatial distribution of RPIs in the Tanshui River estimated by the (**a**) kriging and (**b**) IDW method. Data was obtained at 3 p.m. on 29 September 2010.

**Figure 8:**
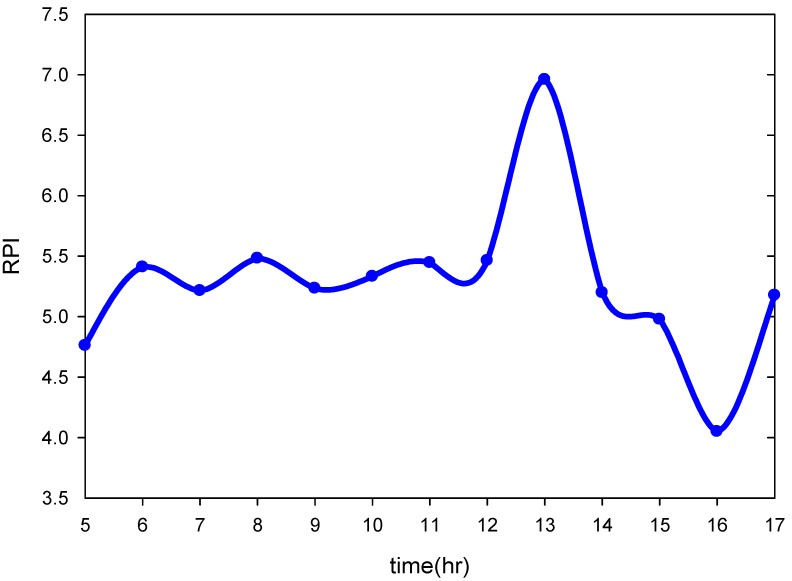
Temporal variation in RPI of the Tanshui River at approximately 23 river kilometers.

Based on Table 1, water quality is classified as unpolluted for the integral of RPI under 2.0; negligibly polluted refers to the integral of RPI between 2.0 and 3.0; moderately polluted indicates water quality above 3.0 but under 6.0. For the integral of RPI above 6.0, water quality is classified as severely polluted. In this study, the RPIs are assigned gradient colors to indicate the levels of river pollution. The estimated RPI from data obtained at 3 p.m. on 29 September 2010, served as the standard value, as shown in Figure 7a. According to the figure, the Tahan River showed the highest pollution level, followed by the Hsintien River; the Keelung River was the least polluted. The water quality of the Tahan River was classified as moderately polluted; the RPI value of the section near the Hsintien River reached 6.83 and was classified as severely polluted. The water quality of the Hsintien River was also classified as moderately polluted; however, the RPI values ranged from 4 to 5, which were lower than that of the Tahan River. Finally, the water quality of Keelung Creek was also classified as moderately polluted; however, the RPI values were largely under 4.3, indicating a lower pollution level than that of the Hsintien River. Moreover, the Tanshui River is a tidal stream, thus allowing the hourly fluctuations during the daytime to be understood through analysis of various RPIs of the river sections. In addition, the inverse distance weighting IDW method was applied for spatial interpolation of RPIs along the Tanshui River, as illustrated in Figure 7b. A comparison of Figure 7a and 7b reveals that if RPI values of adjacent two sampling stations are the same, the RPIs between those two stations remain the same value. However, the RPIs determined through kriging are estimated through the semivariogram *versus* distance, which appears a non-linear relationship in Figure 6. In such a case, the RPIs differ. Moreover, the RPI value estimated by IDW was smaller than that by kriging between Herko and the Guandu Bridge because the RPI at Herko was assumed to be zero. In particular, IDW estimation indicates that the adjacent upstream and downstream river sections are all severely polluted at approximately 23 river kilometers, near the Shinhai Bridge. Figure 8 shows the temporal fluctuations in estimated RPI at the distance. The highest RPI value was apparent at 1 p.m. 

## 5. Conclusions

The water quality of tidal streams was calculated by using conventionally adopted hydrological and water quality models, which are time-consuming and costly. In this study, the pollutant transfer from the upstream to downstream was first estimated by a 1D concept and later used to determine the value of pollutants in a 2D space. The spatial distribution of RPIs of the Tanshui River and its branches was simulated successfully by combining the 1D ordinary kriging method with water quality data collected in the field. This approach is simpler than simulation through conventional 2D variable hydrological models. Moreover, this approach solves the problem of determining complex initial conditions necessary for boundary building in models; instead, only the sampled data are used to represent the average water quality of the studied river section. In this method water quality along a tidal stream can be obtained efficiently. This study also analyzed the spatial distribution of RPIs obtained from various sections at given times in addition to the time distribution for each sampling station. The water quality estimation model in this study was constructed on the basis of the water quality of the tidal stream from the Tanshui River, subsequently allowing for determination of the water quality of various river sections. The results of this study demonstrate the feasibility of using the geostatistics method to estimate the complex water quality of tidal streams.
