# *Correction*: Chen, Y.-C.; Yeh, H.-C.; Wei, C. Estimation of River Pollution Index in a Tidal Stream Using Kriging Analysis. *Int. J. Environ. Res. Public Health* 2012, *9*, 3085-3100

**DOI:** 10.3390/ijerph10062468

**Published:** 2013-06-14

**Authors:** Yen-Chang Chen, Hui-Chung Yeh, Chiang Wei

**Affiliations:** 1 Department of Civil Engineering, National Taipei University of Technology, Taipei 10608, Taiwan; E-Mail: yenchen@ntut.edu.tw; 2 Department of Natural Resources, Chinese Culture University, Taipei 11114, Taiwan; E-Mail: hcyeh@faculty.pccu.edu.tw; 3 Experimental Forest, National Taiwan University, Jhu-Shan, Nantou 55750, Taiwan

The authors wish to add the following amendments and corrections on their paper published in IJERPH [1].

1. Page 3093, line 4, “spherical” and “exponential” were corrected instead of sphere and index. The sentence should read “*The results obtained were then applied to the theoretical semivariogram models, including power, spherical, exponential, and Gaussian models*”. The same situation in page 3095 lines 8, 10, 11 and 12 was also corrected. The sentence should read “*According to Table 3, the coefficients of determination from the spherical, exponential, and Gaussian models were higher than those of the power model. An* inflection phenomenon occurred in the *Gaussian model at the short-distance area, while the spherical model was limited to certain distances. Therefore, in this study, the exponential model was selected to calculate the RPIs for those rivers. Table 4 summarizes the applied results of the exponential model for the 13 h of study along the Tanshui River*”.
2. Page 3093, line 6, “estuaries” should be “tributaries”.
3. Page 3093, Figure 5, we add two river kilometers marked at the conjunction of two tributaries Keelung River and Hsintien River which let the readers acknowledge the exact river kilometers at gauging stations along these two tributaries. The updated Figure 5 should be as follows:

**Figure 5:**
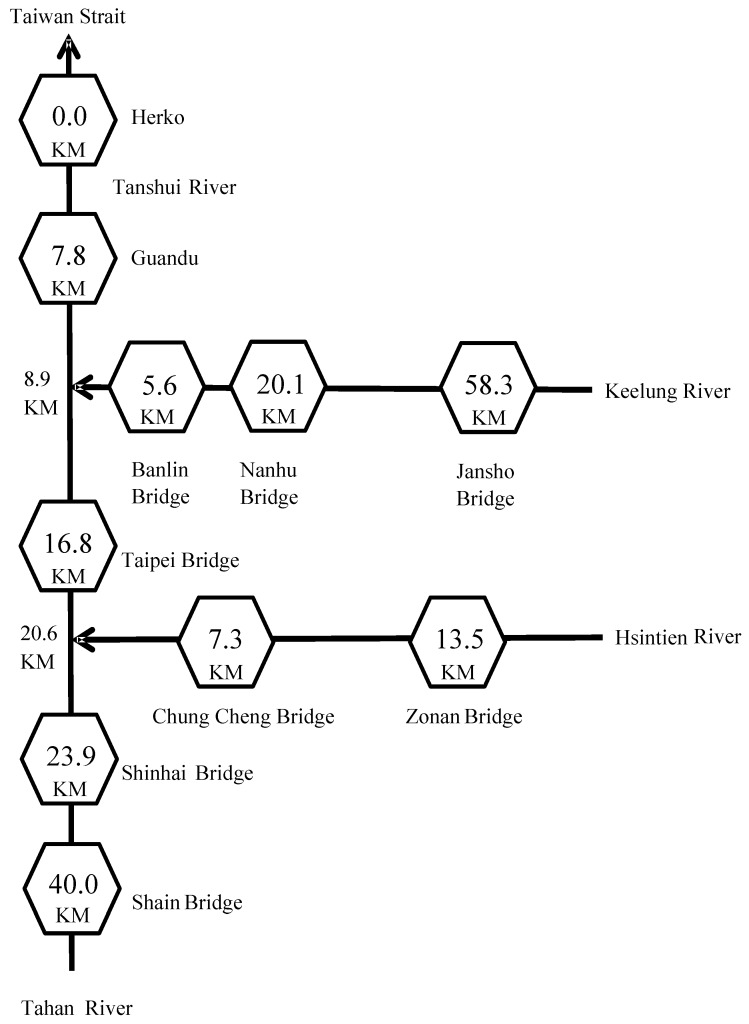
Distances in river kilometers of sampling stations in the catchment of the Tanshui River.
4. Page 3095, line 2, “standard value” should be “starting value”.
5. Page 3095, Table 3, the values of Power and Exponential column were inverse-located. We had corrected this critical error. The updated Table 3 should be as follows:

**Table 3:** Parameters of the four fitted theoretical semivariograms.

Parameter	Power	Exponential	Gaussian	Spherical
*C*_0_	−135.109	−0.005	0.001	−0.006
*c*	136.996	2.318	2.312	2.320
*a*	0.002	0.417	1.000	0.480
Least Error Sum of Squares (RSS)	4.558	3.968	3.968	3.968
Coefficient of Determination (R^2^)	0.4589	0.5289	0.5289	0.5289
6. Page 3096, line 17, “demonstration shown in Figure 7a” instead of “standard value, as shown in Figure 7a”. The sentence should read “*The estimated RPI from data obtained at 3 p.m. on 29 September 2010, served as the demonstration shown in Figure 7a*”.
7. Page 3099 in Acknowledgements, we add another project number of corresponding author and revise “financially/partially supporting” instead of financially supporting. The sentence should read “*The authors would like to thank the National Science Council of Taiwan for financially/partially supporting this research under Contract No. NSC 98-2625-M-027-002 and NSC 97-2313-B-002-044-MY3*”.

The authors apologize for the inconvenience.
